# A new start: *Journal of Animal Science and Biotechnology *re-launches with BioMed Central

**DOI:** 10.1186/2049-1891-3-1

**Published:** 2012-02-28

**Authors:** Defa Li

**Affiliations:** 1 College of Animal Science and Technology, China Agricultural University, Beijing, China

## 

*Journal of Animal Science and Biotechnology *(*JASB*) was founded in June 2010, as the first English language journal published in the field of animal science in China. The journal is supported by the Chinese Association of Animal Science and Veterinary Medicine, a well-respected and established society. Animal science and animal production have been developing rapidly in China over the past 20 years. China has become the largest meat and egg producer in the world. A group of scientists and researchers have emerged in this fast growing area, producing high-quality research results. *JASB *was first designed to introduce achievements in animal science and technology from China to the international community. After publishing several articles, we started to receive excellent submissions from international authors, which made us realize that it was time to move to a truly international platform and make the excellent content visible to readers all over the world. The journal provides a premier platform for your work to reach both Chinese and international audiences. In line with BioMed Central (part of Springer Science+Business Media), the leading Open Access publisher worldwide, the journal is now in an excellent position to make important contributions to the development of animal science and technology research.

Animal Science Research is a diverse program where research areas intersect to study the genetic, nutritional, metabolic, reproductive and physiological mechanisms during animal growth and development, using biotechnology. Animal agriculture continues to expand worldwide, as demand for animal protein and products increase. Research is also conducted in food science and meat science areas to provide consumers with safe, high quality food products. With the continuous development of molecular biology and molecular biotechnology, the reach of the field has become increasingly broad. With the gradual completion of whole-genome sequencing and improvements in biochip technology, the study of heritable changes in gene expression or cellular phenotypes caused by mechanisms, other than changes in the underlying DNA sequence, also becomes a hot topic. At the same, biotechnology applies to animal nutrition research in order to reveal the metabolic mechanism at the molecular level. Traditional animal science research still plays a huge role to improve the quantity and quality of animal products.

Open access is the practice of providing unrestricted access via the Internet to peer-reviewed scholarly journal articles. Open access is increasingly being used to publish journal articles, in addition to theses, scholarly monographs and book chapters [1]. A study published in 2011 on the development of publishing of open access journals from 1993 to 2009 shows that open access journals have increased both in numbers and in average annual output over time [2]. The main reason authors make their articles open access is to maximize the impact of their research [3]. A study in 2001 reported an open access citation impact advantage [4], and lots of studies [5] have confirmed that an open access article is more likely to be read and cited more than one behind subscription barriers. The more the article is read, cited, and built upon, the better for research and for the researcher's career [6,7]. Similarly, the more quickly it is accessible, the better [6]. In most cases, the direct users of research articles are other researchers, open access helps the researchers to read articles more easily. One of the great beneficiaries of open access may be the users in developing countries, where some universities find it is difficult to pay for the required to access the recent journals subscriptions [8].

Since the "birth" of the journal, it has been published under an open access model, allowing all of the publications to be freely accessible online. Springer has been an active player in open access publishing in recent years. Confident in its experience, the journal decided to publish with BioMed Central, the largest open access publisher, and part of Springer. All articles published by *JASB *are made freely and permanently accessible online immediately upon publication, without subscription charges or registration barriers, and, the journal will cover the article-processing charges (APCs) for all authors, so that authors do not need to pay.

*JASB *aims to publish original manuscripts that encompass a broad range of research topics, including animal production and fundamental aspects of genetics, nutrition, physiology, health, quality of animal products, and livestock farming systems. This mainly concerns research in cattle, goats, horses, pigs, and sheep, however, studies involving other farm animals and laboratory animal species that address fundamental issues related to livestock biology will also be considered for publication.

The successful re-launch of *JASB *is the product of many people's hard work. JASB has received lots of help from experts and scholars in field of animal science and biotechnology. I would like to thank you for your continuous support, we are fully committed to conducting thorough peer review and strive to keep the high standards of the journal. We also hope to provide our authors, reviewers and readers a better publishing and reading experience with this new platform. *Journal of Animal Science and Biotechnology *welcomes your best work for rapid open access publication!
